# Gluteal Augmentation with Polymethyl Methacrylate: A 10-year Cohort Study

**DOI:** 10.1097/GOX.0000000000002193

**Published:** 2019-05-31

**Authors:** Roberto Chacur, Honório Sampaio Menezes, Nívea Maria Bordin da Silva Chacur, Danuza Dias Alves, Rodrigo Cadore Mafaldo, Leandro Dias Gomes, Gisele dos Santos Barreto

**Affiliations:** From the * Leger Clinic, Rio de Janeiro, Brazil; † Leger Clinic, São Paulo, Brazil; ‡ Leger Clinic, Porto Alegre, Brazil.

## Abstract

**Background:**

Many techniques for buttocks augmentation have been developed and published, for a more natural, satisfactory, and safe result for the patient. It has been a challenge to find a technique that presented not only volume gain but also gluteal remodeling.

**Methods:**

A total of 1,681 patients who underwent gluteal augmentation with Polymethyl methacrylate (PMMA) between 2009 and 2018 were selected for this retrospective cohort study. Data collected included demographics, procedures data, and postoperative outcomes. Side effects were calculated and compared using the Student’s *t* test.

**Results:**

A total of 1,681 patients (1,583 women and 98 men) who underwent 2,770 gluteal fillings had their cases retrospectively analyzed. They were injected with 540,751.00 mL of PMMA injected. The patients’ mean age was 39 years, and the mean volume injected in each section was 237 mL during the first procedure and 147 mL during the second procedure. The authors observed 52 cases presenting side effects, representing a rate of 1.88% of 2,770 procedures carried out. The statistically significant (*P* = 0.02) presence of side effects was detected in relation to the total filling volume.

**Conclusions:**

This study has demonstrated that gluteal augmentation with PMMA is one of the best options for this type of procedure. In addition, the findings suggest that the guidelines concerning gluteal augmentation must include PMMA filler as an option because PMMA proved to cause few side effects, as demonstrated by this patient cohort.

## Abstract 2

Supplemental Digital Content is available in the text.

## INTRODUCTION

Plastic surgery for improving body contour of the gluteal region has been increasingly sought-after. Badin and Vieira^1^ have described a surgical technique for the placement of high-cohesive round silicone implants using video assistance. Moreover, Jaimovich et al.^2^ have described anchoring sutures, and Sozer et al.^3^ described the use of musculocutaneous flap to increase the buttock in the middle portion and to decrease fat necrosis.

In an attempt to find an ideal surgical technique, Serra et al.^4^ described easily identifiable anatomical landmarks that may assist the surgeon in performing gluteoplasty.

By using a different surgical technique, Sozer et al.^5^ carried out a retrospective study with 10 patients who were submitted to a buttock lift using the skin flap. Patient satisfaction was high, as was in the study conducted by Gonzáles-Ulloa,^6^ who noted a considerable improvement in the postoperative period in relation to patient/surgeon satisfaction.

According to the study by Chacur, it is possible to augment and shape the buttocks using injectable implants with various formulations. Fillers may be used in different regions of the body and face, and in each region, products with different properties may be used, such as PMMA, which is used in large muscle groups.^7^

Lemperle et al.^8^ studied the histological reaction with several substances for filling soft tissues: collagen (Zyplast, Allergan, Irvine, CA), hyaluronic acid (Restylane, Q-med, Uppsala, Switzerland), PMMA microspheres (Artecoll, Canderm Pharma Inc., Canada), silicone oil (PMS 350, Vikomed, Germany), polylactic acid microspheres (New-Fill), dextran microspheres (Reviderm Intra, Medical International, Netherlands), polymethylacrylate (Dermalive, Dermatech, Paris, France), polyacrylamide (Aquamid, Contura – Denmark), polyvinylhydroxide microspheres suspended in acrylamide (Evolution), and calcium hydroxyapatite (FN). The host reacted differently to different fillers; however, all substances, being resorbable or nonresorbable, appeared to be clinically and histologically safe, even though all presented undesirable side effects.

Surgical indications of reconstruction and contouring of the buttocks due to malformation, asymmetry, trauma, and radiotherapy may require corrections made by regular implants, liposuction or lipoinjection, and skin flaps. Buttock implants for aesthetic purposes are widely used, especially in South America. Buttock implants are easy to place and present high success rate, whereas liposuction and lipoinjection procedures require considerable experience of the surgeon in fat injection.^9^

The technique of placement of intramuscular silicone implants provided good results, which resulted in increasing number, consequently, of these procedures in Brazil. However, the data available in the medical literature reveal high rates of wound complications, in particular seromas and dehiscence. According to the study by Serra et al.,^10^ the use of adhesive points and the maintenance of good vascularization in the sacral region are the foundations for reducing complications in gluteoplasty with silicone implants.

According to the study by Chacur, PMMA has been used in medicine for more than 70 years. Among its uses are bone cements, contact and intraocular lenses, bone screw fixation, filling of bone cavities and defects of the skull, and stabilization of vertebrae in patients with osteoporosis or fractures.^7^ Even though there are several new promising alloplastic materials, the versatility and reliability of PMMA allow it to remain a popular and frequently used material.^11^

Hilinski and Cohen^12^ demonstrated improved biocompatibility as a result of increased size and uniformity of PMMA microspheres. This enhanced biocompatibility results in fewer adverse events after the placement of ArteFill (Canderm Pharma Inc, Canada), thus providing a permanent volume increase because the nonabsorbable microspheres stimulate the fibroblasts that synthesize and cause collagen deposition around them. A similar study was also conducted by Mcclelland et al.^13^ The appropriate technique includes deep subcutaneous implantation, with total correction, which is gradually achieved over several treatments. Complications are limited to the formation of nodules, which are easy to handle, and, in most cases, it can be done with conservative interventions.

In a histological study, Lee et al.^14^ claim that the mixture of PMMA and cross-linked dextran in hydroxypropyl methylcellulose can be safely applied to increase soft tissue volume with longevity greater than 12 months. This study demonstrates gluteal augmentation with PMMA and identifies possible side effects and adverse reactions.

## PATIENTS AND METHODS

All procedures performed in this study were in accordance with the ethical standards of the National Commission for Ethics in Research (CONEP), and the 1964 Declaration of Helsinki and its later amendments or similar ethical standards and approved by the ethical committee (CAAE protocol number 86722118.8.0000.5291). Patients were assessed regarding demographics, procedure data, and outcomes. Data were obtained by chart review.

In this retrospective cohort study, cases of 1,681 patients who underwent 2,770 gluteal augmentation with PMMA procedures at the Leger Clinic (in Rio de Janeiro, São Paulo, and Porto Alegre, Brazil) from 2009 to 2018 were analyzed.

There are 3 brands of PMMA allowed in Brazil released by ANVISA (Federal Regulation Agency in Brazil), Biossimetric, MetaDerm (formerly Meta Crill) and Linnea Safe. The ANVISA releases the products for exclusive medical use where the volume varies as required and evaluation.

In this study, gluteal filling with PMMA (Linnea Safe 30% or Meta Crill 30%) is performed under local anesthesia, with the patient awake accompanying by watching the results through a mirror and actively participating in the decisions (**see** video, Supplemental Digital Content 1, which demonstrates a gluteal augmentation technique with PMMA filling, http://links.lww.com/PRSGO/B42).

**Video Graphic 1.**
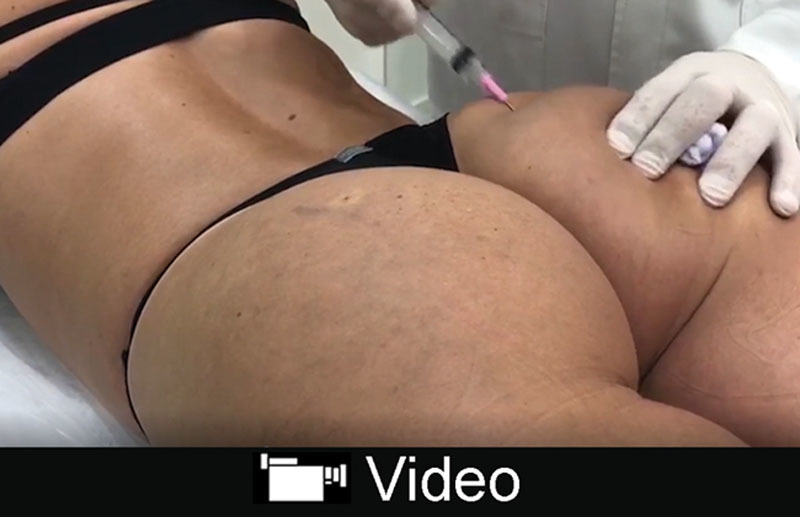
See video, Supplemental Digital Content 1, which demonstrates a gluteal augmentation technique with PMMA filling. This video is available in the “Related Videos” section of the Full-Text article on PRSGlobalOpen.com or available at http://links.lww.com/PRSGO/B42.

The anesthetic and product infiltrations are performed with a 1-mm atraumatic blunt-tipped microcannula, which causes no vascular or nervous lesions in the gluteal muscles and no permanent scarring.

PMMA procedures gluteal filling is contraindicated in a pregnant patient, local infection, systemic infection, local active herpes, autoimmune disease, treatment with immunosuppression, history of keloid formation, history of nodule formation after use of PMMA, use of anticoagulant, in oncologic treatment and history of allergy to the components of the formula.

Student’s t-test was used to verify the data obtained. Analysis of the recorded data took place at the Research Unit of the clinic by using the IBM SPSS Version 22.0 (IBM Corp., Armonk, N.Y.) and the Microsoft Excel (Microsoft Corp., Redmond, Wash.).

## RESULTS

Ninety-eight men (5.8%) and 1,583 women (94.2%) patients had their cases retrospectively analyzed.

Procedures used 540,751.00 mL of PMMA in 1,681 patients. They were submitted to 2.770 gluteal filling sessions, during which 2,002 were performed using Linnea Safe 30% (394,618.00 mL) and 722 using Meta Crill 30% (146,133.00 mL).

The patients’ mean age was 39.31 ± 10.4 years (ranging from 18 to 79 years).

There is no meaningful statistical association between the age group and the occurrence of complications (*P* = 0.291), and age groups are from 18 to 29 years (N = 258; 15.31%), from 30 to 39 years (N = 745; 44.33%), from 40 to 49 years (N = 416; 24.75%), and from 50 to 79 years (N = 262; 15.61%). Most patients were between ages 30 and 39 years (44.33%).

Mean volume per session vary from 237.12 mL on first session to 86.00 mL on last session (Table 1).

**Table 1.**
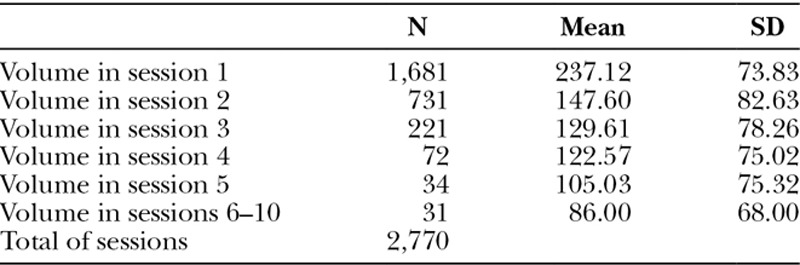
Mean Volume per Session

Only 592 patients had a single application of PMMA (35.21%). More than half of the patients took, on average, 148.91 days (147.85) to have the second procedure performed (Table 2). The delay time between sessions was not related to side effects.

**Table 2.**
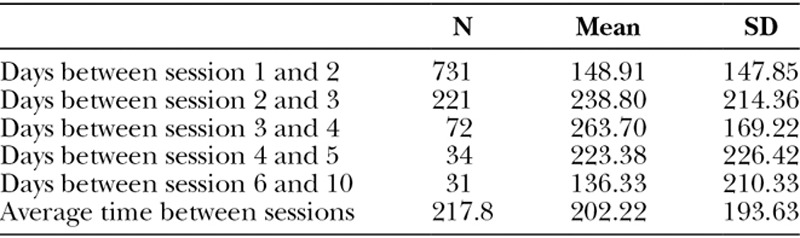
Time Interval (Days) between Sessions

Of a total of 1,681 patients (2.770 procedures), 52 presented side effects, and only 2 patients presented surgical-site infections, representing a rate of 0.07% (Table 3). The most frequent side effects were hematomas (0.36%), seromas (0.29%), and ecchymoses (0.26%). Nevertheless, 98.12% of the procedures presented no side effects. There was no statistically significant difference between the mean age of the patients presenting complications (40.31 years) and the mean age of patients who did not present complications (39.99 years; *P* = 0.783).

**Table 3.**
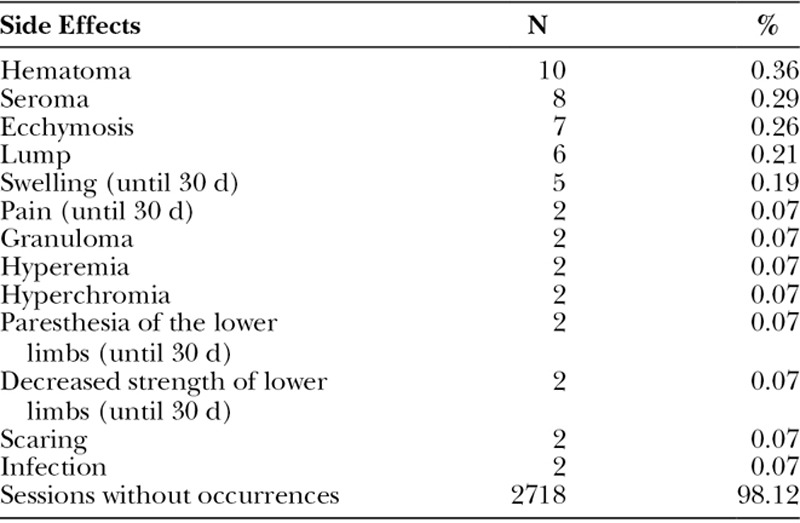
Distribution of the Side Effects in the 2,770 Filling Sessions

There is a statistically significant difference between the mean total volume per session of 24 patients presenting complications (408.42 ± 196.2 mL) and of 1657 patients who did not present complications (326.64 ± 176.26 mL; *P* = 0.024).

In the first session, there was no statistically significant difference between the mean volume per session of patients who presented complications (256.75 mL) and who did not present complications (236.84 mL; *P* = 0.190; Table 4).

**Table 4.**
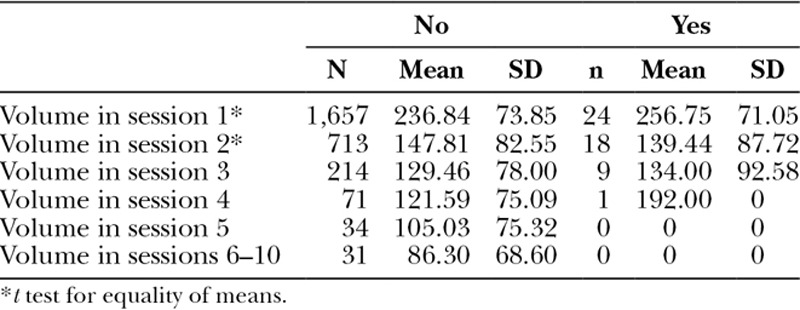
Distribution of the Side Effects in Relation to the Mean Volume Injected in Each Session

Taking under consideration the second session, there was no statistically significant difference between the mean volume per session of patients presenting complications (139.44 mL) and of patients without complications (147.81 mL; *P* = 0.672). Equally, in the third session, there was no statistically significant difference between the mean volume of patients who presented complications (134.00 mL) and those who did not (129.46 mL; *P* = 0.815).

## DISCUSSION

Nowadays, there is a steady increase in the demand for buttock augmentation. Most of the procedures are silicone implant surgeries, which present risks inherent to the technique and the type of the surgical approach, which can be associated with skin flap, liposculpture, and implant placement techniques. Taking all relevant studies from 1980 to 2012 under consideration, Oranges et al.^15^ performed a systematic review on the gluteal augmentation techniques about negative effects on postoperative outcomes of gluteal augmentation techniques.

A study by Vergara and Amezcua presented 160 patients with silicone buttock implants. Thirty patients (18.7%) had implants of 250 cm^3^, 100 (62.5%) received 300 cm^3^ implants, and 30 (18.5%) were implanted with 350 cm^3^ silicone prostheses. There were 16 patients (10%) who presented complications, including seroma in 7 (4%), asymmetry in 4 (2.66%), capsular contracture in 3 (2%), hypercorrection in 1 (0.66%), and rupture of the implant in 1 patient (0.66%).

The volume of the silicone prostheses in the patients in the study by Vergara and Amezcua^16^ is equivalent to the volume in this study. However, the silicone implants presented higher complication rate than the PMMA liquid implant, as shown in Table 3.

Cárdenas-Camarena et al. studied 62 females and 4 males who underwent gluteoplasty in 14 years. Liposuction and lipoinjection were combined. In all cases, liposuction was also performed in other areas.^17^ The infiltrated fat varied from 120 to 280 mL per gluteus muscle, with a mean of 210 mL. Follow-up ranged from 3 months to 3 years and 5 months, with an average of 17 months. Four seromas, 6 visible irregularities, and 2 palpable irregularities occurred among the cases. The complications of lipoinjection occurred in 16 gluteus muscles (12%); all presented temporary hyperemia and erythema, treated with conservative treatments, except in 1 case related to fat necrosis. A probable case of fat embolism syndrome evolved satisfactorily. When compared to the data in this study, in 1,681 patients in 10 years, the index of side effects was only 1.8%. Moreover, there were no cases of necrosis or embolism (Table 3) even though the total injected mean volume was slightly higher (256.75 mL; Table 4).

Oranges et al. reviewed 52 of the most important studies worldwide related to the subject. They all summed up gathered 7,834 patients treated with 5 different gluteal augmentation techniques. The authors characterized the advantages and disadvantages of each technique as follows: procedures with complications (n = 479), 30.5%; liposuction (n = 2,609) with complications, 10.5%; local flap (n = 369) complications, 22%; hyaluronic acid filling (n = 69). These last, which presented no significant complication, even though there was a smaller number of procedures, performed due to the high cost and short duration of its effect.^15^

Results in this study show a significant difference in side effects (1.8%); postoperative surgical-site infections rate was only 0.07%; and other side effects were lower than those registered in Oranges’ review (Table 3) although the technique employed was different from the ones analyzed by the authors (PMMA filling). The surgical-site infection rate in this study was smaller than the common incidence of postoperative surgical-site infections in body contouring surgeries.^18^

Even though gluteoplasties using silicone implants have been performed for decades, wound dehiscence has occurred in 30% of cases.^19^ Such situation does not occur when PMMA is injected because there is no surgical cut.

Serra et al.^20^ determined and quantified the presence of muscle atrophy using computed tomographic scans. All oval-based implants introduced in a vertical direction (7 patients) turned in an oblique direction, 2 patients showed rotation of the implant, and 1 presented muscle atrophy, even though it did not result in clinical or physical limitations. Liquid PMMA does not cause atrophy; on the contrary, it increases the muscle mass. In addition, it does not move or change the position after implantation, which is an advantage in relation to encapsulated silicone implants.

Gluteal augmentation carried out by injecting the patients with volumes from 50 to more than 300 mL of PMMA. Data (Table 4) failed to confirm the general point of view by relating volume session, number of procedures, time between sessions (Table 2), and age of the patients to a higher rate of postoperative surgical site infection, underlining the difficulties of identifying factors that significantly influence the incidence of adverse events following gluteal augmentation with PMMA filler. In addition, the variable of the number of procedures, which is generally accepted as an independent risk factor, could not be significantly related to a higher number of complications in this cohort.^21^

Furthermore, age is not a potentially risk factor because patients older than 50 years are exposed to a 2 times higher risk, approximately, of major complication of a postsurgical surgical-site infection, and in this study, the subjects were under this age. As shown by the data analyzed, the inclusion of PMMA as a standard filler for gluteal augmentation procedures is highly recommended.

Although some studies found differences in the distribution of complications related to sex,^22^ this has not been confirmed by the present study.

Badin and Vieira described a surgical technique for high-cohesive round silicone implants using video assistance. It reduced the risks of sciatic nerve injury in 28 women; moreover, 7% of the complications required reintervention.^1^ In this study, only 2 patients (0.07%) presented local pain for up to 30 days, which may be related to a bundle of sciatic nerve fibers (Table 3).

Serra et al.^4^ described reference anatomical points to study gluteoplasties. The study mentions 1 seroma case, 1 wound infection, and 4 hematomas of the total of 105 cases (3.8%).^4^ It is a low incidence of complications; however, it is still more than double of those found in this study (1.88%; Table 3).

**Fig. 1.**
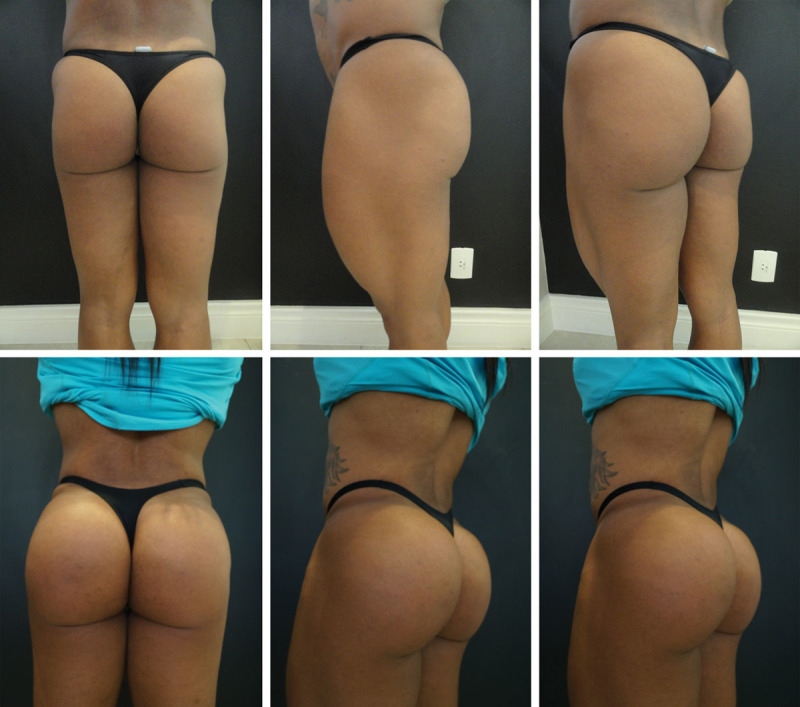
Evolution of PMMA from 1990s to 2000s (from first to third generation). A and B, PMMA of the first generation: spheres of small and irregular size that contribute to the formation of granuloma. C and D, PMMA of the third generation: regular spheres with 40 µm diameter.

One of the great controversies in the use of PMMA is due to the appearance of cases with rejection or displacement of product. However, according to the results described in this study, among 2,770 procedures performed with PMMA of the brands Linnea Safe and Meta Crill there were no cases of rejection, migration, or product displacement. This is because of the physical property of the PMMA, which has solid consistency, and hence, it does not allow migration, and because of its biocompatibility because it is a product used in medicine for more than 70 years in several medical specialties (there are no cases of rejection or allergic processes at this moment). The size of the spheres (40 µm) and their homogeneity due to solid particles and smooth surface help to avoid inflammatory processes (Fig. 1). Moreover, no case of necrosis (due to vascular obstruction) has been observed, since a blunt-tipped atraumatic microcannula is not able to injure blood vessels. Thus, cases that recorded these occurrences are anecdotal and confirm clandestine products on the market. Industrial liquid silicone is the main cause of all the confusion, being responsible for migration, lymphedema, and silicosis.^23^

Adverse effects in this study were seen in 1.88% of the cases (Table 3) with a follow-up of 10 years, much lower than those observed with other techniques, as noted by Oranges et al.,^15^ such as gluteal prostheses (n = 4,781) with complications at 30.5%, liposculpture (n = 2,609) at 10.5%, and local skin flap (n = 369) at 22%.

**Fig. 2.**
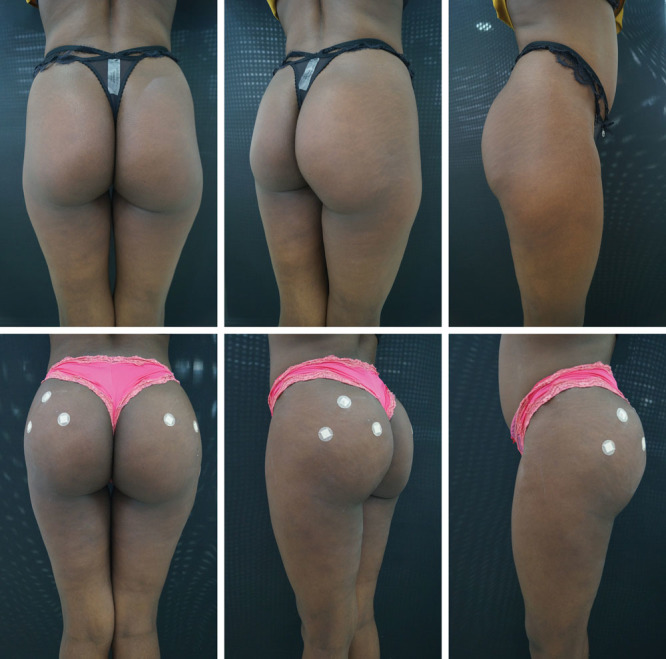
PMMA gluteal filling.7

**Fig. 3.**
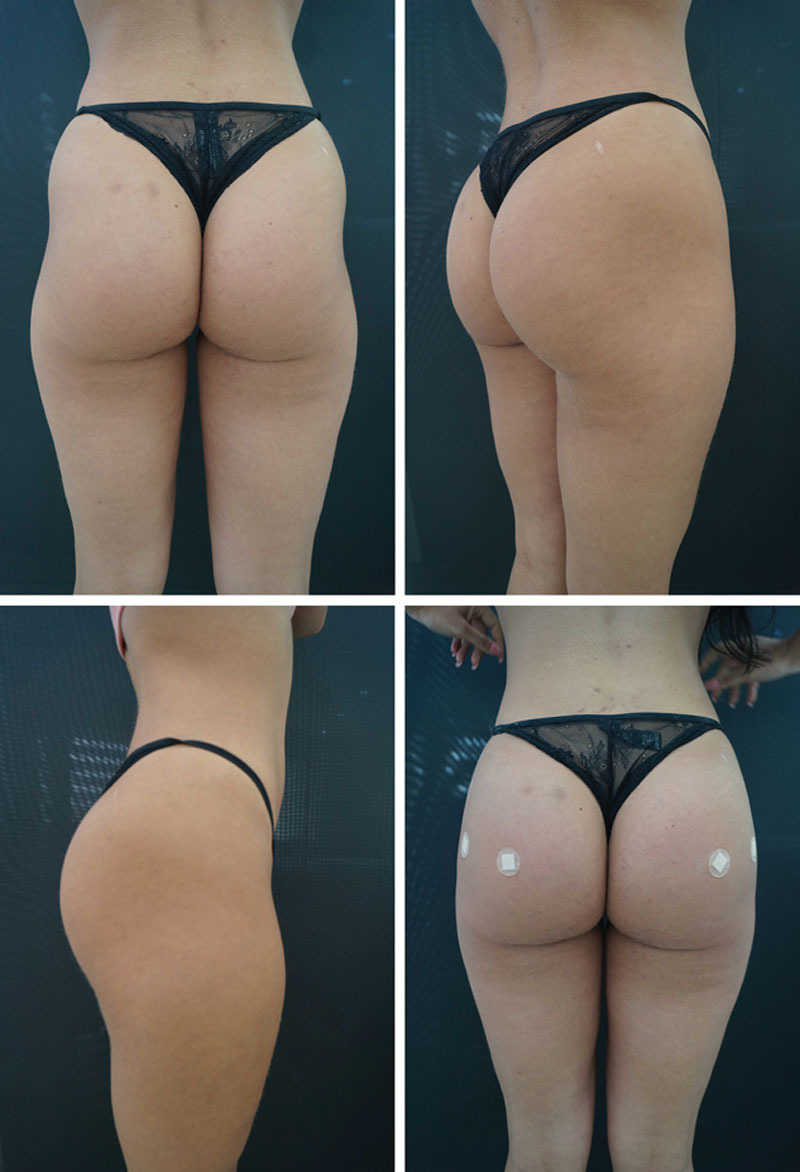
PMMA gluteal filling.

**Fig. 4.**
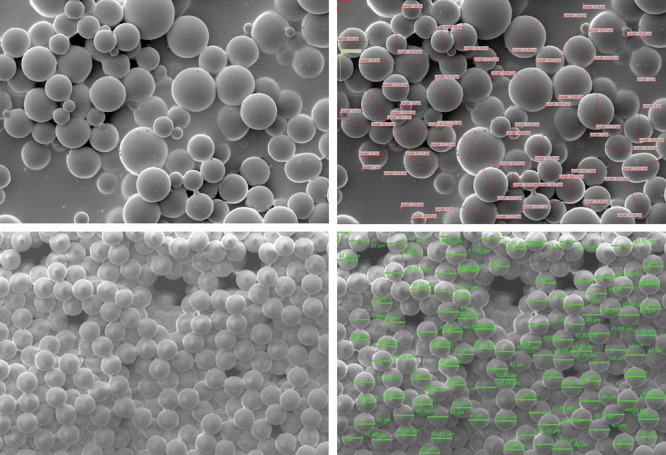
PMMA gluteal filling.

**Fig. 5.**
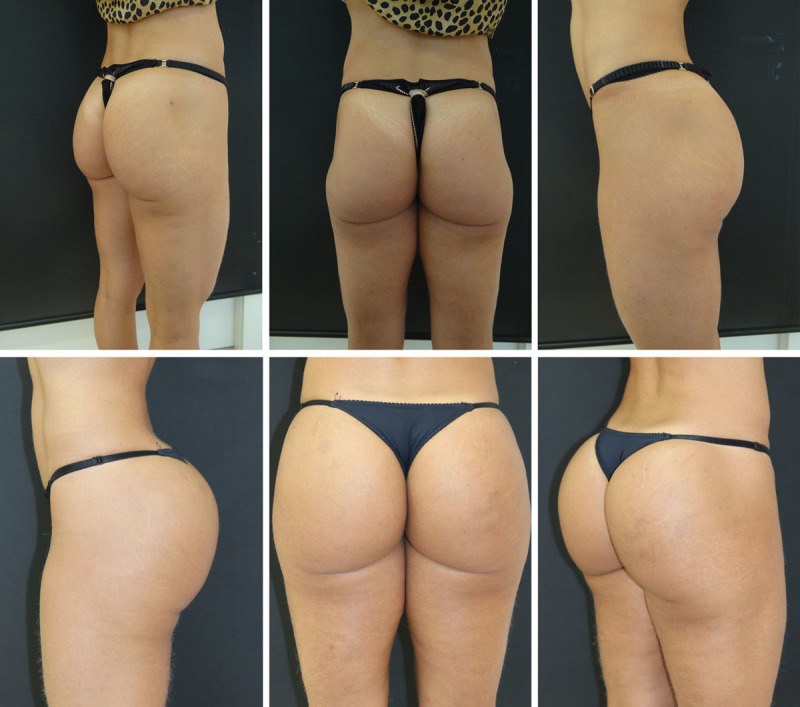
2009 (A) and 2019 (B). Maintenance of the volumetry and improvement in the quality of the skin with lifting effect even with aging.

According to the study by Gonzales,^24^ one of the great challenges in using silicone prostheses, besides the considerable number of complications, is the correction of the format, since the form is considered more important than the volume.^24^ This problem does not exist when using the filling technique described here (Figs. 2–4 and 5).

Based on the 52 complications observed, the cases of seroma and ecchymosis were self-resolving (Table 3). Surgical postoperative treatment was required for only 2 patients who underwent exeresis of a visible palpable nodule in the subcutaneous tissue in an ambulatory surgery under local anesthesia. Possible palpable nodules, which are not visible not even in movement are predicted, and the patients are discouraged by the medical team to have any procedure done.

The low incidence of granuloma is consistent with the current literature^14,25^ where the incidence with purified product (third PMMA generation) fell sharply. In addition, since the product for gluteal augmentation is placed intramuscularly, in the deep plane, it is debatable whether the inflammatory process is the same as the reaction one that occurs in relation to the dermal or subdermal planes. Even if there is an intramuscular granuloma formation in deep muscles, this will be imperceptible to touch.

No cases of late infection or rejection were found in this 10-year follow-up. With PMMA, the result is solid and blood circulation permeates the product, in which infiltrative products, such as intramuscular injections, can be administered. Two cases of infection were found in the immediately after the procedure, which were treated with antibiotic therapy, representing a rate of 0.07%, infinitely lower than that presented by authors of studies carried out with silicone implants, which rates could reach 30.5%.^15^

According to the study by Blanco Souza et al.,^25^ a Brazilian Consensus reached on the use of PMMA. Their trial comprised 87,371 patients treated by several physicians; and 12.285 of these underwent body fillings. The overall complication index of that study was less than 1%, very similar to that found in this study, confirming the safety of the use of PMMA when well applied.

Cárdenas-Camarenas et al.^17^ have studied the cases of 789 patients who underwent gluteal liposuction and lipograft. They were injected with different volumes of fat, varying from 120 to 1,160 mL. Complications, such as fat necrosis, gluteal erythema, infection, and fat embolism syndrome, were more frequent and severe in cases with smaller grafting volume. This was not observed in this study on PMMA injections.

The intramuscular prosthesis placement technique presents high rates of wound complications. Serra et al. studied 20 patients submitted to the gluteal augmentation procedure with the modified technique. This decreased the complication rate of surgical wounds from 35% to 5%, seroma and dehiscence being the most frequent complications.^10^ Even with this reduction, the rate found by the authors was more than twice higher than that found in this study.

Based on the detailed data of patients who underwent procedures and treated postoperatively at our institution, some difficulties that recent studies have encountered could be avoided because the personnel and the protocol at the different centers where the study was carried out were the same. Although Gruskay et al.^26^ reported a significant increase in the absolute number of infections, they estimated a limitation of their findings, since it was a too-large sample size that showed small and, therefore, potentially irrelevant differences. In contrast, results found in this study demonstrate a significant decrease in surgical-site infections in a distinct population of 1,681 patients. Detecting such a difference in our cohort emphasizes the relevance of these findings.

The PMMA in Brazil has regular particles of 40 µm solid diameter and smooth surface. The vehicle and other products already marketed with other raw materials already used are composed of carboxymethylcellulose or hydroxymethylcellulose according to the manufacturer, without anesthetic and without bovine collagen (as BellaFill, a Food and Drug Administration–approved product). The average cost per milliliter in Brazil is U$ 8, attractive cost compared to other surgical techniques, thus allowing the use of this product in large volumes.

Limitations of this study were reduced by a multicenter study design. Thus, data and results could not be potentially biased by specific factors, such as the local medical staff (since it is the same staff) and department environment. This study does not have significant limitations, which makes its results universal and easy to extrapolate.

## CONCLUSIONS

This study has demonstrated that PMMA is one of the best options for gluteal augmentation. Cases of more than 1,600 patients (over 2.770 procedures) were considered, which represents the first demonstration in a large multicenter study that studied the benefits of PMMA filler in gluteal augmentation.

Body contouring surgeries, especially gluteal augmentation, are elective procedures, which make it even more important the postoperative risk assessment, thus further strengthening the significance of this study. In addition, findings suggest that the guidelines concerning gluteal augmentation must include PMMA filler as an option because the substance has been proved to cause few side effects, as demonstrated by this patient cohort.

## ACKNOWLEDGMENTS

The authors thank Dr. Eduardo Luiz da Costa for providing analysis of the PMMA’S performed by LABMIC, Microscopy Laboratory of UFG, Federal University of Goiás (Institute of Physics).

## Supplementary Material
