# Supplementary material for: Gluteal Augmentation with Polymethyl Methacrylate: A 10-year Cohort Study
Source: Plast Reconstr Surg Glob Open. 2019 May 31;7(5):e2193. doi: 10.1097/GOX.0000000000002193 (PMC6571318; doi:10.1097/GOX.0000000000002193)
Supplement: Supplementary file 1 [file gox-7-e2193-s001.doc]

**GLUTEAL AUGMENTATION WITH POLYMETHYLMETACRILATE: A 10-YEAR COHORT STUDY**

**Background:** Many techniques for buttocks augmentation have been developed and published in search for a more natural, satisfactory and safe result for the patient. It has been a challenge to find a technique that presented not only volume gain, but also gluteal remodeling.

**Methods:** 1681 patients who underwent gluteal augmentation with PMMA between 2009 and 2018 were subjects in a retrospective cohort study. Data collected included demographics, procedures data, and postoperative outcomes. Side effects were calculated and compared using the Student’s t-test.

**Results:** 1681 patients (1583 female, and 98 male) who underwent 2770 gluteal fillings had their cases retrospectively analyzed. These comprised 540751 mL of PMMA injected. The patients’ mean age was 39 years, and the mean volume injected in each section was 237 mL during the first procedure, and 147 mL during the second. The authors observed 52 cases presenting side effects, representing a rate of 1.88% of 2770 procedures carried out. A statistically significant (p=0.02) presence of side effects was detected in relation to the total filling volume.

**Conclusions:** This study has demonstrated that gluteal augmentation with PMMA is one of the best options for this type of procedure. In addition, findings suggest that the guidelines concerning gluteal augmentation must include PMMA filler as an option, since the substance proved to cause few side effects, as demonstrated by this patient cohort.

**CLINICAL QUESTION/LEVEL OF EVIDENCE: Therapeutic, III**

**DISCLOSURE:** The authors have no financial interest to declare in relation to the content of this article.

**INTRODUCTION**

Plastic surgery for improving body contour of the gluteal region has been increasingly sought after. Badin and Vieira have described a surgical technique for the placement of high-cohesive round silicone implants using video assistance^1^. Moreover, Jaimovich et al. have described anchoring sutures^2^, and Sozer et al. described the use of musculocutaneous flap to increase the buttock in the middle portion, and to decrease fat necrosis^3^.

In an attempt to find an ideal surgical technique, Serra et al. described easily identifiable anatomical landmarks that may assist the surgeon in performing gluteoplasty^4^.

Using a different surgical technique, Sozer *et al*.carried out a retrospective study with 10 patients who were submitted to a buttock lift using skin flap^5^.Patient satisfaction was high, as was in the study conducted by Gonzáles-Ulloa, who noted a considerable improvement in the postoperative period in relation to patient/surgeon satisfaction^6^.

According to Chacur,it is possible to augment and shape the buttocks using injectable implants with various formulations. Fillers may be used in different regions of the body and face, and in each region products with different properties may be used, such as PMMA, which is used in large muscle groups^7^.

Lemperle et al. studied the histological reaction with several substances (as listed below) used for filling soft tissues^8^. Collagen (Zyplast®), hyaluronic acid (Restylane®), PMMA microspheres (Artecoll®), silicone oil (PMS 350), polylactic acid microspheres (New-Fill®), dextran microspheres (Reviderm® intra), polymethylacrylate (Dermalive®), polyacrylamide (Aquamid®), polyvinylhydroxide microspheres suspended in acrylamide (Evolution®), and calcium hydroxyapatite (FN). The host reacted differently to the fillers; however, all substances, being resorbable or non-resorbable, appeared to be clinically and histologically safe, even though all presented undesirable side effects.

Surgical indications of reconstruction and contouring of the buttocks due to malformation, asymmetry, trauma and radiotherapy may require corrections made by regular implants, liposuction or lipo-injection, and skin flaps. Buttock implants for aesthetic purposes are widely used, especially in South America. These are easy to place, and present high success rate, while liposuction and lipoinjection procedures require considerable experienceof the surgeon in fat injection^9^.

The technique of placement of intramuscular silicone implants provided good results and an increasing number, consequently, of these procedures in Brazil. However, the data available in the medical literature reveal high rates of wound complications, in particular seromas and dehiscence. According to Serra et al., the use of adhesive points, and the maintenance of good vascularization in the sacral region are the foundations for reducing complications in gluteoplasty with silicone implants^10^.

According to Chacur, PMMA has been used in medicine for more than 70 years. Among its uses are bone cements; contact and intraocular lenses; bone screw fixation; filling of bone cavities and defects of the skull; and stabilization of vertebrae in patients with osteoporosis or fractures^7^. Even though there are several new promising alloplastic materials, the versatility and reliability of PMMA allow it to remain a popular and frequently used material^11^.

Hilinskihas demonstrated improved biocompatibility as a result of increased size and uniformity of PMMA microspheres^12^. This enhanced biocompatibility results in fewer adverse events after the placement of ArteFill thus, providing a permanent volume increase, since the nonabsorbable microspheres stimulate the fibroblasts that synthesize and cause collagen deposition around them. A similar study was also conducted by Mcclelland et al.^13^The appropriate technique includes deep subcutaneous implantation, with total correction, which is gradually achieved over several treatments. Complications are limited to the formation of nodules, which are easy to handle, and, in most cases, it can be done with conservative interventions.

In a histological study, Lee et al. claim that the mixture of PMMA and cross-linked dextran in hydroxypropyl methylcellulose can be safely applied to increase soft tissue volume with longevity greater than 12 months^14^. The present study demonstrates gluteal augmentation with PMMA, as well as identifies possible side effects and adverse reactions.

**PATIENTS AND METHODS**

All procedures performed in this study were in accordance with the ethical standards of the National Comission for Ethics in Research (CONEP), and the 1964 Declaration of Helsinki and its later amendments or similar ethical standardsand approved by the Ethical Committee (CAAE protocol number 86722118.8.0000.5291). Patients were assessed regarding demographics, procedure data, and outcomes. Data were obtained by chart review.

In this retrospective cohort study, cases of 1681 patients who underwent 2770 gluteal augmentation with PMMA procedures at the Leger Clinic (in Rio de Janeiro, São Paulo, and Porto Alegre– Brazil) from 2009 to 2018 were analysed.

There are three brands of PMMA allowed in Brasil released by ANVISA (Federal Regulation Agency in Brazil), Biossimetric®, MetaDerm® (former Meta Crill®) and Linnea Safe®. The ANVISA releases the products for exclusive medical use where the volume varies as required and evaluation.

In the present study gluteal filling with PMMA (Linea Safe® 30% or Meta Crill® 30%) is performed under local anesthesia, with the patient awake accompanying by watching the results through a mirror, and actively participating of the decisions (SDC1) (See video, Supplemental Digital Content 1 which demonstrates a gluteal augmentation technique with PMMA filling. THIS VIDEO IS AVAILABLE IN THE “RELATED VIDEOS” SECTION OF THE FULL-TEXT ARTICLE ON PRSJOURNAL.COM OR AT http://links.lww.com/PRSGO/B42)

. The anesthetic and product infiltrations are performed with a1 mm atraumatic blunt-tipped microcanula, which causes novasculho-nervous lesions in the gluteal muscles, and no permanent scarring.

As the most PMMA procedures gluteal filling is contraindicated in a pregnant patient, local infection, systemic infection, local active herpes, autoimmune disease, treatment with immunosuppression, history of keloid formation, history of nodule formation after use of PMMA, use of anticoagulant, in oncologic treatment and history of allergy to the components of the formula.

In order to verify the data obtained, the Student’s t-test was used. Analysis of the recorded data took place at the Research Unit of the clinic by usingthe IBM SPSS Version 22.0 (IBM Corp.®, Armonk, N.Y.), and the Microsoft Excel (Microsoft Corp.®, Redmond, Wash.) softwares.

**RESULTS**

Ninety-eight male (5.8%), and 1583 female (94.2%) patients had their cases retrospectively analysed.

Procedures used 540751.00 mL of PMMA in 1681 patients. They were submitted to 2.770 gluteal filling sessions, during which 2002 were performed using Línea Safe® 30% (394618.00 mL), and 722 using Meta Crill® 30% (146133.00 mL).

The patients’ mean age was 39.31 ± 10.4 years (ranging from 18 to 79 years of age).

There is no meaninful statistical association between the age group in which the patients are and the occurrence of complications (p = 0.291), age groups are from 18 to 29 (N=258; 15.31%), from 30 to 39 (N=745; 44.33%), from 40 to 49 (N=416; 24.75%), from 50 to 79 (N=262; 15.61%). Most patients were between ages 30 and 39 (44.33%).

Mean volume per session vary from 237.12 mL on first session to 86.00 mL on last session (Table 1).

Only 592 patients had a single application of PMMA (35.21%). More than half of the patients took, on average, 148.91 days (147.85) to have the second procedure performed (Table 2). The delay time between sessions was not related to side effects.

Of a total of 1681 patients (2.770 procedures), 52 presented side effects, and only 2, surgical-site infections, representing a rate of 0.07% (Table 3). The most frequent side effects were hematomas (0.36%), seromas (0.29%), and ecchymoses (0.26%). Nevertheless, 98.12% of the procedures presented no side effects. There was no statistically significant difference between the mean age of the patients presenting complications (40.31 years) and the mean age of patients who did not present complications (39.99 years) (p = 0.783).

There is a statistically significant difference between the mean total volume per session of 24 patients presenting complications (408.42 mL ± 196.2), and of 1657 patients who did not present complications (326.64 mL ± 176.26) (p = 0.024).

In the first session there was no statistically significant difference between the mean volume per session of patients who presented complications (256.75 mL), and of patients who did not present complications (236.84 mL) (p = 0.190) (Table 4).

Taking under consideration the second session 2, there was no statistically significant difference between the mean volume per session of patients presenting complications (139.44 mL), and of patients without complications (147.81 mL) (p = 0.672). Equally, in the third session there was no difference between the mean volume statistically significant difference of patients who presented complications (134.00 mL), and those who did not (129.46 mL) (p = 0.815).

**DISCUSSION**

Nowadays, there is a steady increase in the demand for buttock augmentation. Most of the procedures are silicone implant surgeries, which present risks inherent to the technique and to the type of surgical approach, which can be associated with skin flap, liposculpture, and implant placement techniques. Taking all relevant studies from 1980 to 2012 under consideration, Oranges et al.^15^ performed a systematic review on the Gluteal Augmentation Techniques about negative effects on postoperative outcomes of gluteal augmentation techniques.

A study by Vergara et al. presented 160 patients with silicone buttock implants. Thirty patients (18.7%) had implants of 250 cc, 100 (62.5%) received 300 cc implants, and 30 (18.5%) were implanted with 350 cc silicone prostheses. There were 16 patients (10%) who presented complications, including seroma in 7 (4%), asymmetry in 4 (2.66%), capsular contracture in 3 (2%), hypercorrection in 1 (0.66%), and rupture of the implant in 1 patient (0.66%).

The volume of the silicone prostheses in the patients in the study by Vergara et al. is equivalent to the volume in this study^16^. However, the silicone implants presented higher complication rate than the PMMA liquid implant, as shown in Table 3.

Cárdenas-Camarena et al. studied 62 females, and 4 males who underwent gluteoplasties in 14 years. Liposuction and lipoinjection were combined. In all cases, liposuction was also performed in other areas^17^. The infiltrated fat varied from 120 to 280 mL per gluteus muscle, with a mean of 210 mL. Follow-up ranged from 3 months to 3 years and 5 months, with an average of 17 months. Four seromas, six visible irregularities, and two palpable irregularities occurred among the cases. The complications of lipoinjection occurred in 16 gluteus muscles (12%); all presented temporary hyperemia and erythema, treated with conservative treatments, except in one case related to fat necrosis. A probable case of fat embolism syndrome evolved satisfactorily. When comparing to the data in this study, 1681 patients in 10 years, the index of side effects was only 1.8%. Moreover, there were no cases of necrosis or embolism (Table 3), even though the total injected mean volume was slightly higher (256.75mL) (Table 4).

Oranges et al. reviewed 52 of the most important studies worldwide related to the subject. They all summed up gathered 7834 patients treated with 5 different gluteal augmentation techniques. The authors characterized the advantages and disadvantages of each technique as follows: procedures with complications (n = 479) 30.5%; liposuction (n = 2609) with complications 10.5%; local flap (n = 369) complications 22%; and hyaluronic acid filling (n = 69). These last, which presented no significant complication, even though there was a smaller number of procedures, performed due to the high cost and short duration of its effect^15^.

Results in this study show a significant diference in side effects (1.8%); postoperative surgical-site infections rate was only 0.07%; and other side effects were lower than those registered in Oranges’ review (Table 3), although the technique employed was different from the ones analysed by the authors (PMMA filling). The surgical-site infection rate in this study was smaller than the common incidence of postoperative surgical-site infections in body contouring surgeries^18^.

Even though gluteoplasties using silicone implants have been performed for decades, wound dehiscence has occurred in 30% of cases^19^. Such situation does not occur when the technique is injection with PMMA, since there is no surgical cut.

Serra *et al.*determined and quantified the presence of muscle atrophy using computed tomographic scans^20^. All oval-based implants introduced in a vertical direction (7 patients) turned in an oblique direction, two patients showed rotation of the implant, and one presented muscle atrophy, even though it did not result in clinical or physical limitations. Liquid PMMA does not cause atrophy; on the contrary, it increases muscle mass. In addition, it does not move or change position after implantation, which is an advantage in relation to encapsulated silicone implants.

Gluteal augmentation carried out by injecting the patients with volumes from 50mL to more than 300 mL of PMMA. Data (Table 4) failed to confirm the general point of view by relating volume session, number of procedures, time between sessions (Table 2) and age of the patients to a higher rate of postoperative surgical site infection, underlining the difficulties of identifying factors that significantly influence the incidence of adverse events following gluteal augmentation with PMMA filler. In addition, the variable ofthe number of procedures, which is generally accepted as an independent risk factor, could not be significantly related to a higher number of complications in this cohort^21^.

Furthermore, age is not a potentially risk factor because patients older than 50 years are exposed to a two times higher risk, approximately, of major complication of a postsurgical surgical-site infection, and in this study thesubjects were under this age. As shown by the data analysed, the inclusion of PMMA as standard filler for gluteal augmentation procedures is highly recommended.

Although some studies found differences in the distribution of complications related to sex^22^, this has not been confirmed by the present study.

Badin et al. described a surgical technique for high-cohesive round silicone implants using video assistance. It reduced the risks of sciatic nerve injury in 28 women; moreover, 7% of the complications required reintervention^1^. In this study, only two patients (0.07%) presented local pain for up to 30 days**,** which may be related to a bundle of sciatic nerve fibers (Table 3).

Serra et al.^4^ described reference anatomical points to study gluteoplasties. The study mentions one seroma case, one wound infection, and four hematomas of the total of 105 cases (3.8%)^4^. It is a low incidence of complications; however, it is still more than double of those found in this study (1.88%) (Table 3).

One of the great controversies in the use of PMMA is due to the appearance of cases with rejection or displacement of product. However, according to the results described in this study, among 2770 procedures performed with PMMA of the brands Linnea Safe and Meta Crill there were no cases of rejection, migration or product displacement. This is because of the physical property of the PMMA, which has solid consistency, thus, it does not allow migration, and because of its biocompatibility, since it is a product used in medicine for more than 70 years in several medical specialties (there are no cases of rejection or allergic processes at this moment). The size of the spheres (40 microns), as well as their homogeneity, due to solid particles and smooth surface helps to avoid inflammatory processes (Fig 4). Moreover, no case of necrosis (due to vascular obstruction) has been observed, since a blunt-tipped atraumatic microcannula is not able to injure blood vessels. Thus, cases that recorded these occurrences are anecdotal, and confirm clandestine products on the market. Industrial liquid silicone is the main cause of all the confusion, being responsible for migration, lymphedema, and silicosis^23^.

Adverse effects in this study were seen in 1.88% of the cases (Table 3) with a folowup of 10 years, much lower than those observed with other techniques, as noted by Oranges et al.^15^, such as gluteal prostheses (n = 4781) with complications at 30.5%, liposculpture (n = 2609) at 10.5%, and local skin flap (n = 369) at 22%..

According to Gonzales^24^, one of the great challenge in using silicone prostheses, besides the considerable number of complications, is the correction of the format, since the form is considered more important than the volume^24^. This problem does not exist when using the filling technique described here (Fig. 1-2-3, 5).

Based on the 52 complications observed, the cases of seroma and ecchymosis were self-resolving (Table 3). Surgical postoperative treatment was required for only 2 patients which underwent exceresis of a visible palpable nodule in the subcutaneous tissue in an ambulatory surgery under local anesthesia. Possible palpable nodules, which are not visible not even inmovement are predicted, and the patients are discouraged by the medical team to have any procedure done.

The low incidence of granuloma is consistent with the current literature ^14,25^ where the incidence with purified product (3th PMMA generation) fell sharply. In addition, since the product for gluteal augmentation is placed intramuscularly, in the deep plane, it is debatable whether the inflammatory process is the same as the reactional one that occurs in relation to the dermal or subdermal planes. Even if there is intramuscular granuloma formation in deep muscles, this will be imperceptible to touch.

No cases of late infection or rejection were found in this 10-year follow-up. With PMMA the result is solid, blood circulation permeates the product, in which infiltrative products, such as intramuscular injections, can be administered. Two cases of infection were found in the immediate post-procedure, which were treated with antibiotic therapy, representing a rate of 0.07%, infinitely lower than that presented by authors of studies carried out with silicone implants, which rates could reach 30.5%^15^.

According to Souza et al., a Brazilian Consensus reached on the use of PMMA^25^. Their trial comprised 87371 patients treated by several physicians; and 12.285 of these underwent body fillings. The overall complication index of that study was less than 1%, very similar to that found in the present study, confirming the safety of the use of PMMA when well applied.

Cárdenas-Camarenas et al.^17^ have studied the cases of 789 patients who underwent gluteal liposuction and lipograft. They were injected with different volumes of fat, varying from 120 to 1160 mL. Complications, such as fat necrosis, gluteal erythema, infection, and fat embolism syndrome were more frequent and severe in cases with smaller grafting volume. This was not observed in the present study on PMMA injections.

The intramuscular prosthesis placement technique presents high rates of wound complications. Serra et al. studied 20 patients submitted to the gluteal augmentation procedure with the modified technique. This decreased the complication rate of surgical wounds from 35% to 5%, being the most frequent complications seroma and dehiscence^26^. Even with this reduction, the rate found by the authors was more than twice higher than that found in this work.

Based on the detailed data of patients who underwent procedures and treated postoperatively at our Institution, some difficulties that recent studies have encountered could be avoided, since the personell and the protocol at the different centers where the study was carried out were the same. Although Gruskay et al. reported a significant increase in the absolute number of infections; they estimated a limitation of their findings, since it was a too-large sample size that showed small and, therefore, potentially irrelevant differences^27^. In contrast, results found in this study demonstrate a significant decrease in surgical site infections in a distinct population of 1681 patients. Detecting such a difference in our cohort emphasizes the relevance of these findings.

The PMMA in Brazil has regular particles of 40 microns solid diameter and smooth surface. The vehicle, as well as other products already marketed with other raw materials already used, is composed of carboxymethylcellulose or hydroxymethylcellulose according to the manufacturer, without anesthetic and without bovine collagen (as BellaFill has, a FDA approved product). The average cost per mL in Brazil is U$ 8, thus allowing the use of this product in large volumes and attractive cost compared to other surgical techniques.

Limitations of this study were reduced by a multicenter study design. Thus, data and results could not be potentially biased by specific factors, such as the local medical staff (since it is the same staff), and department environment. This study does not have significant limitations, which makes its results universal and easy to extrapolate.

**CONCLUSIONS**

This study has demonstrated that PMMA isone of the best options for gluteal augmentation. Cases of more than 1600 patients (over 2.770 procedures) were considered, which represents the first demonstration in a large multicenter study that studied the benefits of PMMA filler in gluteal augmentation.

Body contouring surgeries, especially gluteal augmentation, are elective procedures, which make it even more important the postoperative risk assessment, thus, further strengthening the significance of this study. In addition, findings suggest that the guidelines concerning gluteal augmentation must include PMMA filler as an option, since the substance has been proved to cause few side effects, as demonstrated by this patient cohort.

**ACKNOWLEDGMENTS**

The authors thank Dr. Eduardo Luiz da Costa for providing analysis of the PMMA'S performed by LABMIC - Microscopy Laboratory of UFG - Federal University of Goiás (Institute of Physics).

Roberto Chacur, MD

Research Unit

Leger Clinic

Rio de Janeiro, Brazil

roberto.chacur@clinicaleger.com.br

**REFERENCES**

1. Badin AZ, Vieira JF. Endoscopically assisted buttocks augmentation. Aesthetic Plast Surg. 2007 Nov-Dec; 31 (6):651-6.

2. Jaimovich CA, Almeida MW, Aguiar LF, da Silva ML, Pitanguy I. Internal suture technique for improving projection and stability in secondary gluteoplasty. Aesthet Surg J. 2010 May-Jun; 30(3):411-3.

3. Sozer SO, Agullo FJ, Palladino H. Split gluteal muscle flap for autoprosthesis buttock augmentation. Plast Reconstr Surg. 2012 Mar; 129 (3):766-76.

4. Serra F, Aboudib JH, Cedrola JP, de Castro CC. Gluteoplasty: anatomic basis and technique. Aesthet Surg J. 2010 Jul-Aug; 30 (4):579-92.

5. Sozer SO, Agullo FJ, Palladino H. Autologous ugmentation gluteoplasty with a dermal fat flap. Aesthet Surg J. 2008 Jan-Feb; 28 (1):70-6.

6. González-Ulloa M. Gluteoplasty: a ten-year report. Aesthetic Plast Surg. 1991 Winter; 15 (1):85-91.

7. Chacur R. Ciência e Arte do Preenchimento. 1 ed. Porto Alegre: AGE, 2018, 262p.

8. Lemperle G, Morhenn V, Charrier U. Human histology and persistence of various injectable filler substances for soft tissue augmentation. Aesthetic Plast Surg. 2003 Sep-Oct; 27 (5):354-66.

9. Harrison D, Selvaggi G. Gluteal augmentation surgery: indications and surgical management. J Plast Reconstr Aesthet Surg. 2007; 60 (8):922-8.

10. Serra F, Aboudib JH, Marques RG. Reducing wound complications in gluteal augmentation surgery. Plast Reconstr Surg. 2012 Nov; 130 (5):706e-713e.

11. Frazer RQ, Byron RT, Osborne PB, West KP. PMMA: an essential material in medicine and dentistry. J LongTermEff Med Implants. 2005; 15 (6):629-39.

12. Hilinski JM, Cohen SR. Soft tissue augmentation with ArteFill. Facial Plast Surg. 2009 May; 25(2):114-9.

13. Mcclelland M, Egbert B, Hanko V, Berg RA, DeLustro F. Evaluation of artecoll polymethylmethacrylate implant for soft-tissue augmentation: biocompatibility and chemical characterization. Plast Reconstr Surg. 1997 Nov;100 (6):1466-74.

14.Lee YB, Park SM, Song EJ, Park JG, Cho KO, Kim JW, Yu DS. Histology of a novel injectable filler (polymethylmethacrylate and cross-linked dextran in hydroxypropyl methylcellulose) in a rat model. J Cosmet Laser Ther. 2014 Aug;16 (4):191-6.

15. Oranges MC, Tremp M, Summa GP, Haug M, Kalbermatten FD, Harder Y, Schaefer JD. Gluteal Augmentation Techniques: A comprehensive Literature Review. Aesthetic Surgery Journal. 2017 feb 37(5) 560-569

16. Vergara R, Amezcua H. Intramuscular gluteal implants: 15 years' experience. Aesthetic Surg J 2003; 23:86-91.

17. Cárdenas-Camarena L, Arenas-Quintana R, Robles-Cervantes JA Plast Buttocks fat grafting: 14 years of evolution and experience. Reconstr Surg. 2011 Aug;128(2):545-55.

18. Cruse PJ, Foord R. A five-year prospective study of 23,649 surgical wounds. Arch Surg. 1973:107-206–210.

19. Mendieta CG. Gluteoplasty.Aesthet Surg J. 2003 Nov-Dec; 23 (6):441-55.

20. Serra F, Aboudib JH, Marques RG. Intramuscular technique for gluteal augmentation: determination and quantification of muscle atrophy and implant position by computed tomographic scan. Plast Reconstr Surg. 2013 Feb; 131(2):253e-259e.

21. Duscher D, Kiesl D, Michael M et al. Seasonal impact on surgical-site infections in body contouring surgery: retrospective cohort study of 602 patients over a period of 6 years. Plast Reconstr Surg 2018:142(3):653-660.

22. Chong T, Coon D, Toy J, Purnell C, Michaels J, Rubin JP. Body contouring in the male weight loss population: Assessing gender as a factor in outcomes. Plast Reconstr Surg. 2012;130:325e–330e.

23. Almeyer DM, Anderson LL, WangAR. Silicone migration and granuloma formation. J Cosmet Dermatol 2009: 8, 92-97

24. Gonzales R. Intramuscular Gluteal Augmentation: The XYZ Method. Clin Plastic Surg. 2018 (45) 217-223.

25. Souza T, Colomé L, Bender E, Lemperle G. Brazilian Consensus Recommendation on the Use of Polymethylmetacrylate Filler in Facial and Corporal Aesthetics. Aesth Plast Surg. 2018 jun:1-8

26. Serra F, Aboudib JH, Marques RG. Reducing wound complications in gluteal augmentation surgery. Plast Reconstr Surg. 2012 Nov;130(5):706e-713e.

27. Gruskay J, Smith J, Kepler CK, et al. The seasonality of postoperative infection in spine surgery: Clinical article. J Neurosurg Spine 2013;18:57–62

FIGURES

Figure 1- PMMA gluteal filling.

Figure 2- PMMA gluteal filling.

Fig 3- PMMA gluteal filling.

Figure 4 – Evolution of PMMA from 90`s to 2000`s (from 1th to 3th generation).

Figure 4 A-B – PMMA of the first generation: spheres of small and irregular size that contribute to the formation of granuloma.

Figure 4 C-D – PMMA of the third generation: regular spheres with 40µm diameter.

Figure 5 – 2009(before) and 2019(after) . Maintenance of the volumetry and improvement in the quality of the skin with lifting effect even with aging.

See video, Supplemental Digital Content 1 which demonstrates a gluteal augmentation technique with PMMA filling. This video is available in the “related videos” section of the full-text article on prsjournal.com or at insert link here
